# A survey on women’s awareness of iron and folic acid intake during preconception period and its associated factors in Manna District, Oromia region, Southwest Ethiopia

**DOI:** 10.1002/nop2.1041

**Published:** 2021-08-20

**Authors:** Firanbon Teshome, Yohannes Kebede, Kasahun Girma, Zewdie Birhanu

**Affiliations:** ^1^ Department of Health, Behavior and Society Faculty of Public Health Jimma University Jimma Ethiopia

**Keywords:** care, nutrition, pregnancy

## Abstract

**Aim:**

This study aimed to assess pregnant women's awareness of iron and folic acid intake during the preconception period and associated factors.

**Design:**

A cross‐sectional study.

**Methods:**

This study was conducted on 636 pregnant women in Manna District, Oromia region, Southwest Ethiopia. Women's awareness of iron and folic acid intake during the preconception period was measured using a pre‐tested structured questionnaire. Descriptive, binary and multivariable logistic regression analyses were carried out.

**Results:**

Of the total of 623 participants, 6.7% of them had an awareness of iron and folic acid intake during preconception period. Women's husband who had formal education, women who had ≥four ANC visits and women who were at distance of <30 min from the nearest health facility were significant predictors of pregnant women's awareness iron and folic acid intake during the preconception period.

**Conclusions:**

Women's awareness of iron and folic acid intake during the preconception period was very low. Husband educational status, frequency of ANC visits and distance from health facility were predictors of women's awareness of iron and folic acid intake during the preconception period.

## 1 INTRODUCTION

Despite the global target is to reduce anaemia in women of reproductive age by 50% by 2025 (Targets, 2014), it is still high, especially in developing countries and difficult to attain this target. Evidence showed that, globally, anaemia affects 29% of pregnant women and 38% of non‐pregnant women (Stevens et al., 2013). Also, in Ethiopia, about 24% of reproductive age group women (Rahman et al., 2016) and 31.7% of pregnant women were anaemic (Kassa et al., 2017). Folic acid (FA) deficiency is also among the major public health problems, especially among reproductive age group women. Worldwide, more than a third of women are estimated to have folic acid deficiency (Ferreira & Gama, 2010). Similarly, the prevalence of folic acid deficiency is also high among the Ethiopian reproductive age group women, and in that, 46% of them had severe foliate deficiency (Haidar et al., 2010).

Iron and folic acid deficiencies are the most important public health problems causing adverse pregnancy outcomes like maternal and foetal morbidity and mortality (Black et al., 2008; Rahman et al., 2016). For example, the consequences of anaemia and iron deficiency are multiple and serious, affecting not only the health of individuals, but also the development of societies and countries. In low‐ and middle‐income countries, anaemia contributes to 18% of perinatal mortality, 19% of preterm births and 12% of low birth weight (Rahman et al., 2016). On the other hand, deficiency of folic acid during preconception care is main risk factor for the occurrence of neural tube defects (NTDs) like an anencephaly, encephalopathy and spinal bifida (Biermann et al., 2006; Lima et al., 2009; Lu et al., 2006). NTDs are the second most common birth defect after congenital cardiac malformations (Cheschier, 2003). It has been reported that more than 300,000 cases of NTDs occur worldwide each year, and many are in low‐income countries, including Ethiopia (de Andrade Silva Cavalcanti, 2018).

In Ethiopia, hydrocephalus (35.5%), neural tube defects (27.5%) and neuro‐trauma (9.7%) were the three major leading causes of admission to hospitals for surgical procedures among children (Tadesse et al., 2014). These defects in turn have a major impact on the child, families and societies. Almost all babies born with anencephaly die soon after birth. Babies born with spinal bifida have a 10‐fold higher risk of death during their childhood compared to those without spinal bifida in developed countries (Kancherla et al., 2014; Wang et al., 2011). This risk of death is expected to be much higher in developing countries, where access to the healthcare services is limited. Additionally, these defects cause negative consequences like physical challenges (paralysis of the legs, bowel and bladder management problems), mental challenges (mental retardation, educational, emotional issues, self‐esteem), social challenges for life and economic problems (Al‐Holy et al., 2013; Grosse et al., 2016). Folic acid deficiency is also associated with hypertensive pregnancy syndrome, recurrent spontaneous abortions, premature births, low birth weight, chronic cardiovascular and vascular brain diseases, dementia and depression (Lima et al., 2009; Xu et al., 2014).

Most of the iron and folic acid deficiencies that lead to the above‐mentioned adverse outcomes happen in reproductive‐aged group women were because of inadequate intake or low absorption of iron, loss of iron through menstruation and frequent pregnancies (American College of Obstetricians and Gynecologists, 2008). To overcome such challenges, supplementation of iron and folic acid is recommended for childbearing age group women before conception. For instance, the WHO recommends that all women of childbearing age consume 400 μg of folic acid daily and that women with pregnancies previously affected by NTDs consume 5,000 μg of folic acid daily at least one‐month prior conception (World Health Organization, 2012). There are also recommendations on iron supplementation for women of childbearing age groups. The WHO 2009 recommended that, in population groups where the prevalence of anaemia is >20% among women of reproductive age and mass fortification programmes of staple foods with iron and folic acid are unlikely to be implemented within 1–2 years, weekly iron‐folic acid supplementation (60 mg iron in the form of ferrous sulphate and 2,800 μg folic acid) should be considered as a strategy for the prevention of iron deficiency, the improvement of pre‐pregnancy iron reserves and the improvement of folate status (World Health Organization, 2009). The WHO 2016 also recommended daily iron supplementation (30–60 mg/daily for the duration of three consecutive months) as a public health intervention for women of childbearing age, living in settings where anaemia is highly prevalent (≥40% anaemia prevalence; World Health Organization, 2016). Combining iron with folic acid prior to and during pregnancy has benefits (Gardiner et al., 2008). In addition to supplementations, women should also eat foods rich in iron and folate like brown rice, red meat, liver, poultry, egg yolk, legume, dark‐green leafy vegetables, citrus fruits, beans, and peas, both before and during pregnancy (Brognoli, 2010; Dietary Guidelines Advisory Committee, 2010; Pontes et al., 2008).

On top of these recommendations, there was evidence that showed the adverse effects of iron and folic acid deficiencies can be reduced or prevented by giving great attention to the women's health throughout the continuum of maternal and child health, especially during the preconception period. For examples, studies indicated that the prevalence of neural tube defects was decreased by 50%–70% through supplementation of folic acid during the preconception period (Chen et al., 2008; Salih et al., 2014). Thus, good health and nutrition (like iron and folic acid) before conception are key to a mother's ability to meet the nutrient demands of pregnancy and breastfeeding and are vital to the healthy development of the embryo, foetus, infant, and child (Hanson et al., 2015). However, despite its importance and recommended by WHO (World Health Organization, 2009, World Health Organization, 2012, World Health Organization, 2016), to the best of the authors’ knowledge, there are no published articles on iron and folic acid use during the preconception period in Ethiopia. Therefore, this study aimed to assess pregnant women's awareness of iron and folic acid intake during preconception period and associated factors in the Manna district, Oromia region, Southwest Ethiopia, 2019.

### 1.1 Research questions

The following research questions guided our study:

- What is the magnitude of women's awareness of iron and folic acid intake during preconception period?
- What are factors associated with women's awareness of iron and folic acid intake during preconception period?

## 2 THE STUDY

### 2.1 Aim of the study

This study aimed to assess the magnitude of women's awareness of iron and folic acid intake during preconception period and its associated factors.

### 2.2 Study design

A community‐based cross‐sectional study was carried out from 02 March 2019 to 10 April 2019.

### 2.3 Study setting

The study was conducted in the Manna district among pregnant women. The Manna district is one of the 21 districts found in Jimma zone, Oromia Region. It is located 368 km southwest far from Addis Ababa and 22 km from Jimma town. According to the 2019 report obtained from the Manna District Health Office, the district has a total population of 197,911, of which 26,451 were urban and 171,460 were rural. Women of reproductive age groups of the district were 43,738, and pregnant women were 6,868. The district has a total of 26 kebeles: 1 urban and 25 rural kebeles. It has 7 health centres, 26 health posts, 11 private clinics and 3 private pharmacies. It has also 68 health extension workers and 121 healthcare providers of different professions.

### 2.4 Participants and sampling

The source populations were all pregnant women found in the district during the study period, and the study populations were randomly selected pregnant women who fulfilled the inclusion criteria. All pregnant women who lived in the district for at least six months prior to the study period were included in the study. Pregnant women who were critically ill and unable to communicate were excluded.

The sample size was determined by using a single population proportion formula, considering the following assumptions: 50% proportion of women's awareness of iron and folic acid intake during the preconception period, since there was no prior study in Ethiopia specifically to address the study objectives, 95% level of confidence, 5% margin of tolerable sampling error, 10% non‐response and 1.5 design effects. Based on these, the final sample size of the study was 636. In order to select the study participants, first, the 26 kebeles were stratified into rural and urban. Then, the urban kebele was included in the study purposively for representation. Eight kebeles among the 25 rural kebeles were selected using a simple random sampling technique. Then, the sample size was proportionally allocated to the selected 9 kebeles. The lists of the total number of pregnant women found in the selected rural kebeles were obtained from the family folder of the community health information system, which is available at the health post. For the urban kebele, since the family folder did not exist, a census was conducted to construct the sampling frame. Finally, computer‐generated simple random sampling was used to identify the study participants. Their usual place of residence was identified in collaboration with kebele leaders.

### 2.5 Data collection

Data were collected using an interviewer‐administered structured questionnaire adapted from different studies and modified to the local context. It was first prepared in English and then translated to Afan Oromo and Amharic by experts. Then, it was translated back to English by another person to ensure its consistency and accuracy. The questionnaire has four parts: socio‐demographic and economic characteristics (12 items), obstetric and gynaecologic history (9 items), pre‐existing medical illness (3 items) and awareness of iron and folic acid intake during preconception period (4 items). A pretest was conducted among 5% of pregnant women in the Saka district, which is located 20 km away from the study area. A total of 6 data collectors (4 clinical nurses and 2 BSc nurses) and 2 public health officers as supervisors were recruited based on their previous experience in data collection and fluency in the languages of the community. In addition, the authors also closely supervised the data collection processes. The data collectors and supervisors were trained for one day on the objective of the study, data collection tool, approach to the interviewees, details of interviewing techniques, respect and maintaining privacy and confidentiality of the respondents. Reliability analysis showed that the Cronbach's α coefficient of the tool was 0.88 and acceptable (α ≥ .70), indicating the tool was reliable (Bolarinwa, 2015).

### 2.6 Study variables, operational definitions and measurement

Awareness of iron and folic acid intake during preconception period was the dependent variable, and socio‐economic and demographic factors (age, residence, educational level, occupation, marital status, family size and wealth of the household), gynaecologic and obstetric factors (history of family planning use prior to conception, pregnancy planning status, parity, gravidity and ANC visit), pre‐existing medical illnesses, health facility‐related factor (distance from health facility) and media‐related factors (Radio, Television) were independent variables.

#### 2.6.1 Awareness

In this study, awareness is defined as having ever “heard” or “read” about iron and/or folic acid intake during preconception period for the sake of becoming pregnant (Al‐Holy et al., 2013; Rofail et al., 2012).

#### 2.6.2 Preconception period

There is no a single consensus on the definition of preconception period. Some scholars defined it as “a minimum of one year prior to the initiation of any unprotected sexual intercourse” (Dean et al., 2013). Others defined as three months before conception (Gnoth et al., 2003; Potter & Parker, 1964). The discrepancy of definitions was due to the fact that some preconception health improvements take weeks (e.g. achieving adequate folate concentrations through supplements) while others taking months or years (e.g. achieving a healthy weight). In this study, preconception period is defined as a period of a least three months before conception occurs.

### 2.7 Data analysis

After checking the completeness of the data manually, the collected data were entered, cleaned and checked using Epi data Manager Version 4.0.2. Then, the data were exported to SPSS version 21 for analyses. Bivariable and multivariable logistic regression analyses were carried out to identify an association between the predictors and outcome variables. Binary logistic regression analysis was performed to select variables for multivariable logistic regression analysis. Variables with a *p*‐value <.25 in the binary logistic regression analysis were taken as candidates for multivariable logistic regression analysis. Finally, multivariable logistic regression analysis was performed to control for the possible confounding effects of the selected variables. Variables with a *p*‐value <.05 were recognized as statistically significant associations with women's awareness of preconceptional iron and folic acid use. Odds ratio with its 95% CI was used to show the degree of association between the independent and outcome variables. Descriptive analyses like frequencies and proportions were also conducted for different variables as necessary.

### 2.8 Ethical consideration

A letter of Research Ethics Committee was received from the Institutional Review Board of Jimma University. The necessary permission was obtained from Manna district health office and kebele administrative offices. All the study participants were informed about the purpose of the study, their right to refuse and assured about the confidentiality of the information they provided. Their informed consent was obtained prior to the interview.

## 3 RESULTS

### 3.1 Participant demographics

A total of 623 pregnant women participated, giving a response rate of 98.0%. More than half of the respondents, 352 (56.5%) and 328 (52.6%), were in the age range of 25–34 years and had no formal education respectively. A majority of the respondents, 553 (88.8%), 583 (93.6%) and 462 (74.2%), lived in rural areas, Muslims by religion, and housewives in their main occupation respectively (Table 1).

**TABLE 1:** Socio‐demographic characteristics of the respondents (*N* = 623)

Variable	Category	*N* (%)
Age	15–24	196 (31.5)
	25–34	352 (56.5)
	35–49	75 (12.0)
Residence	Rural	553 (88.8)
	Urban	70 (11.2)
Religion	Muslim	583 (93.6)
	Orthodox	28 (4.5)
	Protestant	12 (1.9)
Educational status	No formal education	328 (52.6)
	Primary education (1–8)	231 (37.1)
	Secondary education (9–12)	56 (9.0)
	Tertiary (college or university)	8 (1.3)
Husband education	No formal education	298 (47.8)
	Primary education (1–8)	253 (40.6)
	Secondary education (9–12)	60 (9.6)
	Tertiary (college or university)	12 (1.9)
Occupation of the respondents	Housewife	462 (74.2)
	Farmer	106 (17.0)
	Merchant	39 (6.3)
	Other^a^	16 (2.6)
Marital status	Married	618 (99.2)
	Other^b^	5 (0.8)

Kaffa, Gurage and Silxe.

^a^ Student, daily worker, private employee and government employee.

^b^ Single and separated.

### 3.2 Participants obstetric and gynaecologic history, and pre‐existing medical Illness

Of the total of 623 respondents, 98 (15.7%) of the women had become pregnant for the first time. A majority, 369 (59.2%), 421 (67.6%) and 423 (67.9%) of the women, had gestational age of 13–28 weeks, multiparous and planned pregnancy respectively (Table 2).

**TABLE 2:** Obstetric and gynaecologic, and pre‐existing medical illness of the respondents (*N* = 623)

Variable	Category	*N* (%)
Gestational age	≤12 weeks	43 (6.9)
	13–28 weeks	369 (59.2)
	29–36 weeks	209 (33.5)
	≥37 weeks	2 (0.3)
Gravidity	Prim gravida	98 (15.7)
	Multigravida	525 (84.3)
Parity	Null parous	103 (16.5)
	Prim parous	99 (15.9)
	Multiparous	421 (67.6)
ANC visit	Not on ANC visit	217 (34.8)
	One visit	45 (7.2)
	Two visits	130 (20.9)
	Three visits	99 (15.9)
	Four or more visits	132 (21.2)
Pregnancy planning status	Planned	423 (67.9)
	Unplanned	200 (32.1)
Pre‐existing medical illness	Yes	46 (7.4)
	No	577 (92.6)

### 3.3 Women's awareness of preconceptional iron and folic acid intake

Findings showed that, of 623 participants, only 38 (6.1%) and 26 (4.2%) of them had awareness of iron and folic acid intake during the preconception period respectively. Forty‐two (6.7%) of the participants had awareness of intake of at least one of the two micronutrients (iron or folic acid) during the preconception period.

### 3.4 Initial source of information for preconceptional iron and folic acid intake

**FIGURE 1:**
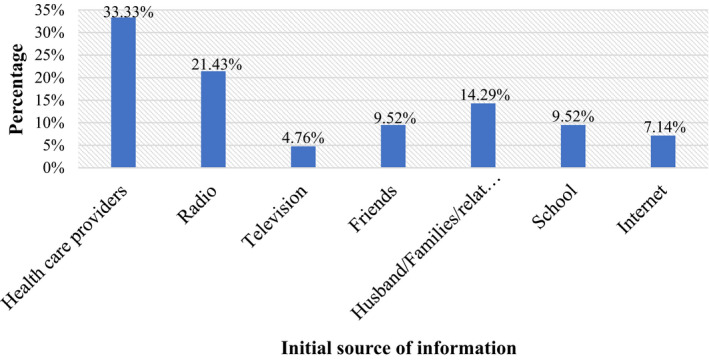
Initial source of information of iron and folic acid intake during the preconception period

Of the total respondents who had awareness of preconceptional iron and folic acid intake, their initial sources of information for one out of three (33.33%) participants were healthcare providers (Figure 1).

### 3.5 Factors associated with women's awareness of iron and folic acid intake during preconception period

Findings of multivariable logistic regression analysis showed that the odds of having awareness of iron and folic acid intake during the preconception period were nearly three times higher for women whose their husbands had formal education and women who were at distance of <30 min from the health facility compared to their counterparts [AOR = 2.84, 95% CI: (1.15–7.03)] and [AOR = 2.83; 95% CI: (1.23–6.55)] respectively. Women who had four or more ANC visits were 2.31 times more likely to have awareness of iron and folic acid intake during the preconception period compared to those who had <4 ANC visits [AOR = 2.31; 95% CI: (1.01–5.26)] (Table 3).

**TABLE 3:** Predictors of women's awareness of iron and folic acid intake during the preconception period in Manna district, Jimma zone, Oromia region, Southwest Ethiopia, 2019 (*N* = 623)

Variable	Awareness of Fe and FA		COR [95% C.I]	AOR [95% C. I]
	Yes	No		
Women's educational status				
Had formal education	25	270	1.69 [0.90–3.20]	0.82 [0.33–2.0]
No formal education	17	311	1.00	1.00
Husband educational status				
Had formal education	33	292	3.63 [1.71–7.72]	2.84 [1.15–7.03]^a^
No formal education	9	289	1.00	1.00
Pregnancy planning status				
Planned	35	388	2.49 [1.09–5.70]	1.62 [0.52–5.07]
Not planned	7	193	1.00	1.00
ANC visits				
<4	14	260	1.00	1.00
≥4	16	116	2.56 [1.21–5.42]	2.31 [1.01–5.26]^a^
Wealth index				
Low	11	203	1.00	1.00
Middle	8	192	0.77 [0.30–1.95]	0.77 [0.23–2.63]
High	23	184	2.31 [1.09–4.86]	1.17 [0.42–3.26]
Distance from health facility				
<30 min	23	203	2.25 [1.20–4.24]	2.83 [1.23–6.55]^a^
≥30 min	19	378	1.00	1.00
Media				
Had media (TV/Radio)	25	239	2.10 [1.11–3.98]	0.85 [0.36–2.01]
No Media (TV/Radio)	17	342	1.00	1.00

Abbreviations: FA, folic acid; Fe, iron; Hx, history.

^a^ Statistically significant at *p*‐value <.05.

## 4 DISCUSSION

The finding of this study showed that the magnitude of women's awareness of iron and folic acid intake during the preconception period was 6.7%. Women's husband who had formal education, those who had four or more ANC visits, and at distance of <30 min from the nearest health facility were significant predictors of pregnant women's awareness iron and folic acid intake during the preconception period.

The results of the current study revealed that only 6.7% of the participants had awareness of iron and folic acid intake during preconception period. This finding was far lower than studies done in developed countries like Western Australia (89%; Oddy et al., 2007), USA (88%; Sharp et al., 2009), Norway (60%; Daltveit et al., 2004), Lebanon (60%; Hage et al., 2012), Spain (50.6%; Coll et al., 2004), Turkey (48.2%; Köken et al., 2013), United Arab Emirates (46.4%; Abdulrazzaq et al., 2003), Honduras (46.4%; Milla et al., 2007), Europe (40%; Bitzer et al., 2013) and China (36%; Ren et al., 2006). The reasons for this difference might be due to media access in previous studies. This might also due to the difference in the study population. Majority of the previous studies were conducted among women of reproductive age groups and postpartum women. However, our study was conducted among pregnant mothers. The other possible explanation for this difference might be due to the previous studies were facility‐based. However, our study was community‐based which the representation of the true population is. People who attended health facilities might have more information about the preparations needed and services provided before conception. In addition to this, the lowest awareness in the current study might also be due to majority of the study participants in the current study had no formal education, even though education is a source of information. Indeed, the variation might also be due to the difference in healthcare system between the current study and previous studies.

The finding of this study was also lower than studies conducted in developing countries like Saudi Arabia (88.4%; Al‐Akhfash et al., 2013), Thailand (76.1%; Nawapun & Phupong, 2007), Egypt (71.6%; Hassan et al., 2015), Nigeria (64.6%; Anzaku, 2013) and Adet Northwestern Ethiopia (15.9%; Goshu et al., 2018). This might be due to the previous studies were facility‐based, and some of them conducted among urban women. However, our study was community‐based and the majority (88.8%) of the participants were from rural areas, which is the representation of the true population. Women who attend health facilities and live in urban might have awareness about health issues and provided services due to their higher chance of getting information. This implies the importance of conducting an awareness creation intervention. However, the finding of the current study was in line with studies conducted in Pakistan (6.7%; Rehan et al., 2015), Tanzania (6.9%; Mwandelile et al., 2019) and Nepal (5%; Paudel et al., 2012). The slight difference might be probably due to time variation between the current study and previous studies.

Our study also identified predictors of women's awareness of iron and folic acid use during preconception period. In this study, Husbands educational status determined women's awareness of preconceptional iron and folic acid intake. Women whose husbands had formal education were nearly three times more likely to have awareness of iron and folic acid intake during preconception period than their counterparts. This might be due to the fact that in the study area, husbands who had formal education were higher than women who had formal education, and probably women might get information about health issues from their husbands. The other possible reason might be that, in the study area, husbands were the only decision maker among the family members in all activities. This makes the women difficulty of attending health facilities and different meetings or conferences. Educated husbands might have information about maternal and child health issues, have joint plan discussions with their partner and support their spouse to get information about health and services given before and during pregnancy (Abrha et al., 2020). This implies the importance of improving the educational status of the communities.

The frequency of ANC visits was also among the predictors of women's awareness of preconceptional iron and folic acid use. Women who had four or more ANC visits were 2.3 times more likely to have awareness of preconceptional iron and folic acid use than those who had less than four ANC visits [AOR = 2.31; 95% CI: (1.01–5.26)]. This finding was similar to studies conducted in Nigeria (Lawal & Adeleye, 2014), Sudan (Alsammani et al., 2017) and Hawasa South Ethiopia (A. Kassa & Yohannes, 2018). The similarity might be due to the fact that contact with healthcare providers increases the chance of obtaining information about health issues and services. As the number of contacts with healthcare providers increases, the chance of obtaining information also increases, which in turn increases the health seeking behaviour and service utilization. In current study, distance from health facilities determined women's awareness of iron and folic acid use the during preconception period. Women who were at distance of <30 min from the nearest health facility were more likely to have awareness of iron and folic acid intake during the preconception period compared to those who were at distance of ≥30 min. This might be due to the fact that as communities are at large a distance from health facilities, it is less likely for them to contact healthcare providers, which in turn decreases their probability of obtaining information about their health issues. This might also be due to the fact that in the study area that health education is usually given only at health facilities, and outreach campaigns like awareness creation campaigns were not well practised. This makes people far from health‐related information.

### 4.1 Limitations of the study

A study does not end without limitations. Recall bias may occur on some questions such as those related to obstetric and gynaecologic factors as pregnant women were asked for history before they conceived. In this study, pregnant women were recruited instead of women in preconception period due to budget constraint, as list of pregnant women wase easily obtained from the family folder of the community health information system. Interviewer bias may also have occurred. However, to mitigate this, one day intensive training was given to both data collectors and supervisors on the objective of the study, data collection tool, approach to the interviewees, details of interviewing techniques, maintaining privacy and confidentiality of the respondents.

## 5 CONCLUSION

In this study, women's awareness of preconceptional iron and folic acid use was very low. Husband educational status, frequency of ANC visits and distance from health facility were significant predictors of women's awareness of iron and folic acid intake during the preconception period. Therefore, healthcare providers are advised to counsel women attending health facilities for different services like family planning, antenatal care and postnatal care on the importance of taking iron and folic acid supplements, the proper time, and food rich in iron and folic acid. In addition, healthcare providers are also recommended to conduct awareness creation interventions for communities. Indeed, media should also give more emphasis on providing information about iron and folic acid. Moreover, we advise researchers to conduct studies on related issues among different women of reproductive age groups such as women planning to become pregnant and currently married women using strong study designs.

## CONFLICT OF INTEREST

The authors declare that they have no conflict of interests.

## AUTHOR CONTRIBUTION

FT, YK and ZB: Conceptualization, design, data collection, analysis, report writing, interpretation, writing drafts of the manuscript and critical revision of the drafts. KG: Analysis, interpretation, writing and critical review of the drafts. Authors read and approved the final manuscript and agreed for submission.

## ETHICS APPROVAL AND CONSENT TO PARTICIPATE

A letter of Research Ethics Committee was received from the Institutional Review Board of Jimma University. In addition, the official letter of cooperation was obtained from Manna district health office. The necessary permission was obtained from kebele leaders. All the study participants were informed about the purpose of the study, their right to refuse and assured about the confidentiality of the information they provided. Their informed consent was obtained prior to the interview.

## CONSENT FOR PUBLICATION

Not applicable.

## DATA AVAILABILITY STATEMENT

The data of the study are available from the corresponding author on reasonable request.
